# Does delay in planned diabetes care influence outcomes for aboriginal Australians? A study of quality in health care

**DOI:** 10.1186/s12913-019-4404-7

**Published:** 2019-08-19

**Authors:** Shu Qin Li, Steven Guthridge, Paul Lawton, Paul Burgess

**Affiliations:** 1 Northern Territory Department of Health, PO Box 40596, Casuarina, NT 0811 Australia; 2 Menzies School of Health Research, PO Box 41096, Casuarina, NT 0811 Australia

**Keywords:** Diabetes, Chronic disease, Care plan, Quality of care, Optimal care, Outcomes, Northern Territory

## Abstract

**Background:**

To examine the association between delay in planned diabetes care and quality of outcomes.

**Methods:**

A retrospective analysis of primary care and inpatient records for 2567 Aboriginal patients, with diabetes, living in 49 remote communities in the Northern Territory of Australia. Poisson regression was used to estimate the association between delay from diagnosis to documented diabetes care plan and three outcome measures: mean HbA1c level, most recent blood pressure and number of diabetes-related hospital admissions.

**Results:**

Compared with no delay (< 60 days), patients with delay had increased risk of elevated mean HbA1c: 60 days to < 2 years, incidence rate ratio (IRR), 1.2 (95% CI:1.07–1.39); 2 years to < 4 years, incidence rate ratio (IRR), 1.2 (95% CI:1.04–1.45); 4 years and over, incidence rate ratio (IRR), 1.3 (95% CI:1.12–1.52). There was no evidence of association between delay and optimal blood pressure control. Risk of diabetes-related admission increased with increased delay. Compared with no delay the IRRs for delay were: 60 days to < 2 years, 1.2 (95% CI:1.07–1.42); 2 to < 4 years, 1.3 (95% CI: 1.15–1.58): and 4 years and over, 2.6 (95% CI,2.28–3.08).

**Conclusion:**

The study found that a timely diabetes care plan was associated with better short-term blood glucose control and fewer diabetes-related admissions but not with improved blood pressure control. Delays may be a result of both patient and service-related factors.

**Electronic supplementary material:**

The online version of this article (10.1186/s12913-019-4404-7) contains supplementary material, which is available to authorized users.

## Background

Diabetes mellitus is a major health problem in Australia, estimated to affect 5.1% of the adult population [1] and, in 2008–2009, to cost $A1507 million in health care. [2] For Aboriginal and Torres Strait Islander peoples (hereafter referred to as Aboriginal people) diabetes is even more prevalent and has greater impact. A recent national biometric survey included 3300 Aboriginal participants, aged 18 years and over, and estimated that the diabetes prevalence was 11.0%, more than twice the prevalence in the general population. [3] Within the Northern Territory (NT) Aboriginal population the survey results indicated a gradient in diabetes prevalence from 9.4% in urban areas to 20.8% in remote areas, which is consistent with a previous NT remote community study. [4] Diabetes, in association with complications such as cardiovascular and renal disease, is a major cause of premature mortality and the associated 10 year gap in life expectancy between Aboriginal and non-Aboriginal Australians. [5]

Primary care is the first point of contact for patients with diabetes, and adherence with recommended primary care management has been demonstrated to slow the progression of diabetes complications among Aboriginal Australians and to be cost effective. [6–9] One NT study described a U-shaped association between utilisation of primary care and hospital admissions, with groups of Aboriginal patients with diabetes and moderate levels of primary care utilisation having fewer total hospital admissions than low or high utilisation groups. [10] However utilisation alone does not indicate whether a patient receives recommended care. A key measure of the quality of chronic disease management, including diabetes, is whether a patient has a documented patient-specific care plan. [11] A chronic disease care plan typically contains a general practitioner (GP) led holistic assessment of patient comorbidities and social functioning, patient self-management goals and treatment preferences, clinical treatment goals, an individually tailored annual program of care and monitoring and team care arrangements allocating tasks to patients’ care providers consistent with evidence-based guidelines. [10, 12, 14, 15] A previous study demonstrated that a General Practice Management Plan for diabetes patients resulted in significant improvement in clinical tests and outcomes including HbA1C level, cholesterol level and body mass index (BMI). [13] A number of international studies have also reported that the presence of patient-specific diabetes care plans result in increased care seeking activities such as increased blood glucose tests and foot and eye care, a short term improvement in glycaemic control and reduced diabetes related hospital admissions. [16–19] In the context of the implementation of diabetes care plans, one area for which there has been no previous reports is the effect of a delay in the introduction of care plans on the quality of clinical outcomes.

In the NT, there has been a sustained focus on improved management of chronic diseases, including diabetes, in the Aboriginal population. [20] This has included the introduction of electronic health records that provide decision support for standardised care for clients with diabetes. To date, there has been limited assessment of the impact of planned patient care for diabetes, indicated by the presence of a diabetes care plan in Aboriginal populations [21, 22]. The aim in this study is to examine the association between varying delays between diagnosis and the documentation of a diabetes care plan and the quality of clinical management.

## Method

### Data sources

Two data sources were used to identify all Aboriginal clients, aged 15 years or older, with a diagnosis of diabetes during the study period from 1 Jan 2008 and 30 June 2013. The Primary Care Information System (PCIS) contains electronic health records for approximately 30,000 NT Aboriginal clients attending 49 health centres operated by the NT Department of Health in remote NT communities. The NT Hospital Separations Dataset (HSD) contains comprehensive information on admissions to all five NT public hospitals.

The NT Department of Health uses a unique identifier, known as the Hospital Registration Number (HRN), for all client related services across the Department. The HRN has been used within the department for more than 20 years and is maintained within a central data warehouse with a program of continuing review and consolidation. The HRN has been used in a number of previous linkage studies and has been reported as highly reliable. [23, 24] In the present study we linked unit-level data within the PCIS and HSD datasets, using the HRN, for all individuals with a diagnosis of diabetes.

### Case definition

In PCIS clinical conditions are coded using the International Classification of Primary Care, revised second edition (ICPC-2-R). [25] The study cohort was selected from PCIS records on the basis of any of three criteria: an ICPC-2-R diagnosis code for diabetes (Additional file 1: Table S1); a pharmacy record for insulin; or, a pathology record of a glycated haemoglobin (HbA1C) result greater than 6.5%. The HSD includes information on primary diagnosis and up to 49 secondary diagnoses for all admissions, coded using the International Classification of Disease 10th Version, Australian Modified (ICD-10-AM). [26] Cases were selected from the HSD if a patient had an address within the service areas of the 49 health centres and a record for hospital admission with either a primary or secondary diagnosis of diabetes.

### Outcome variables

Our study examined the relationship between a delay from diagnosis to the documentation of a diabetes care plan and three outcome measures – mean Haemoglobin A1c (HbA1c) level, latest recorded blood pressure (BP) and number of diabetes-related hospital admissions.

HbA1C is an indicator of blood glucose control in the three-months before the test. [13] The cut-off point between prediabetes and diabetes is a HbA1C of 6.5%, and in practice a result of 7% or lower indicates good diabetes control. For each patient in the study from one to 18 measurements were available to estimate a mean HbA1C level. We used a cut-off of a mean HbA1C level of 7% and over as a binary outcome for poor blood glucose control. Renal disease is a common diabetes complication and blood pressure control is a key intervention to slow the progress of disease [27]. We used the most recent blood pressure result and the recommended cut-off points for “normal” blood pressure of a systolic BP of 130 mmHg and diastolic BP of 80 mmHg as a binary outcome of optimal control. The number of diabetes-related hospital admissions was used as a continuous variable, with a range from 0 to 63 admissions.

### Explanatory variables

The key explanatory variable was the delay between diagnosis and documentation of a diabetes care plan. The date of the first recorded diagnosis was extracted from PCIS or HSD datasets. This variable of “delay” was grouped into four categories: no delay (less than 60 days); from 60 days to less than two years; from two years to less than four years; and, four years or greater. Two other time related variables were developed for use in multivariate analyses. PCIS was implemented over several years and completed by 2011. [8, 9] At the time of implementation, in each health centre, information from historical patient records was uploaded including the dates of diagnosis of diseases and dates of implementation of care plans. It is possible that historical data was either incomplete or not entered accurately. To address this uncertainty, a binary variable was created as to whether a diagnosis of diabetes was made before or after the date of implementation of electronic primary care records.

Two demographic variables, age group (in ten year groups) and sex, were included in the analyses to accommodate variations in diabetes incidence and health seeking behaviour.

The two administrative health service regions in the Northern Territory were included in the analysis - the Central Australian region and the northern Top End region.

### Statistical analysis

Multivariate Poisson regression was used to examine the association between categories of delay and the three outcome measures with results expressed as incidence rate ratio (IRR). The models included adjustment for age, sex, time-in-study and whether the diagnosis occurred before or after the implementation of electronic records.

## Results

The study population was 2567 patients, including 946 men (36.9%) and 1621 women (63.1%). The distribution of category of delay in the documentation of a diabetes care plan for the 2567 patients with primary care data is presented, by sex, in Table 1. There was a similar proportion of men (26.3%) and women (26.0%) with no delay. Of all patients, 670 (26.1%) had no delay; a further 600 patients (23.4%) had a delay from 60 days to less than two years; 317 patients (12.3%) had a delay from two years to less than four years; and 915 patients (35.6%) had a delay of at least 4 years before a documented care plan. Patients who had a delay of more than four years tended to have a younger median age at diagnosis in both men and women. A small number (65 or 2.6%) of patients, with primary care data, had no record of care plan at the end of the study period. Most of this group were recently diagnosed and were not included in multivariate analyses.

**Table 1:** Distribution of age and sex in patients with diabetes by levels of delay in development of care plan, NT Aboriginal population, 2008–2013

	Men			Women		
Delay in care plan	Number	Percent	Median age	Number	Percent	Median age
No delay	249	26.3	43.5	421	26.0	40.9
60 days to < 2 years	214	22.6	42.3	386	23.8	42.3
2 to < 4 years	119	12.6	44.1	198	12.2	41.7
4 + years	333	35.2	40.9	582	35.9	39.0
No care plan	31	3.3	41.8	34	2.1	38.6
Total	946	100.0	42.4	1621	100.0	40.5

There was strong evidence of an association between any delay in implementation of a care plan and an HbA1c above the recommended range for 1626 patients with documented results (Table 2). After controlling for age, sex and timing of the introduction of electronic health records, patients with a delay of 60 days and less than two years had an IRR of 1.2 for having a mean HbA1C above the recommended level compared with patients with no delay. Similarly the risks were greater for those patients with a delay of 2 years to less than 4 years (IRR 1.2) and 4 or more years (IRR 1.3). The results for BP control for 754 patients with documented results are also presented in Table 2 and provide no evidence of an association between delay in implementation of a diabetes care plan and quality of blood pressure control.

**Table 2:** The association between delay in development of care plans and optimal HbA1C level and blood pressure control in patients with diabetes, NT Aboriginal population, 2008–2013

HbA1C				
Delay in care plan	No	Mean	IRR (95%CI)	*P*-value
No delay	414	7.5	Reference	
60 days to < 2 years	367	8.5	1.2 (1.07–1.39)	0.002
2 to < 4 years	220	8.3	1.2(1.04–1.45)	0.025
4 years and over	625	8.7	1.3 (1.12–1.52)	0.001
Sex (men v women)			0.9 (0.84–0.97)	0.007
Age group (ref: 15–24 years)			1.0 (0.99–1.0)	0
Period before e-records			1.02 (0.90–1.15)	0.81
Blood pressure (BP)				
	No	(Mean BP: S/D)	IRR (95%CI)	*P*-value
No delay	177	127/80	Reference	
60 days to < 2 years	165	127/79	0.96 (0.87–1.07)	0.52
2 to < 4 years	120	129/80	0.98 (0.85–1.12)	0.75
4 years and over	292	128/77	0.92 (0.81–1.05)	0.23
Sex (men v women)			1.17 (1.10–1.24)	0.00
Age group (ref: 15–24 years)			1.00 (1.00–1.01)	0.01
Period before e-records			1.03 (0.92–1.15)	0.61

Note: *S* Systolic, *D* Diastolic

There was a wide range in the number of diabetes-related hospital admissions for individual patients in the study population and an apparent gradient in the mean number of admissions of men and women with each category of increasing delay in development of a care plan (Table 3). This gradient is in part explained by those patients with categories of increasing delay in implementation of care plans also having, on average, longer time in the study. Among both men and women there was a mean of 0.7 admissions for those with no delay increasing to means of 4.0 and 3.5 admissions, respectively, for those with four or more years delay. The gradient was evident in both the Top End and Central Australian health service regions, with hospital admissions more common in all categories of delay for the study population from Central Australia.

**Table 3:** Mean number of diabetes-related hospital admissions for patients with diabetes by varying length of delay in development of care plans, by sex and region, NT Aboriginal population, 2008–2013

	Men		Women	
Delay in care plan	Mean	Range	Mean	Range
No delay	0.7 (249)	(0,35)	0.7 (421)	(0,15)
60 days to < 2 years	1.5 (214)	(0,31)	1.3 (386)	(0,25)
2 to < 4 years	1.4 (119)	(0,22)	2.0 (198)	(0,43)
4 + years	4.0 (333)	(0,45)	3.5 (582)	(0,63)
No care plan	2.2 (31)	(0,22)	1.7 (34)	(0,19)
Total	2.2		2.0	
	Top End Region		Central Australian Region	
Delay in care plan	Mean	Range	Mean	Range
No delay	0.6 (385)	(0,13)	0.9 (285)	(0,35)
60 days to < 2 years	1.3 (315)	(0,31)	1.4 (285)	(0,25)
2 to < 4 years	1.4 (175)	(0,19)	2.3 (142)	(0,43)
4 + years	3.5 (450)	(0,63)	3.9 (465)	(0,45)
No care plan	2.2 (36)	(0,19)	1.6 (29)	(0,22)
Total	1.9		2.4	

The results of Poisson regression of the association between delay in development of a care plan and number of diabetes-related admissions, adjusted for sex, age and timing of the introduction of electronic health records are presented in Table 4. The results demonstrate evidence of an increase in mean number of diabetes-related admissions with delay in implementation of a care plan. Patients with delays of from 60 days to less than 2 years were 1.2 times more likely to be hospitalised compared with patients with no delay. Similarly those patients with a delay between 2 and less than 4 years were 1.30 times more likely to be hospitalised and those with a delay of 4 or more years were 2.6 times more likely to be hospitalised compared with patients who had no delay. There was a strong evidence that being diagnosed with diabetes before the implementation of electronic records in primary care were associated with increased risk of hospital admission.

**Table 4:** The association between delay in the development of care plans and number of diabetes-related hospital admissions for patients with diabetes, NT Aboriginal population, 2008–1013

Delay in care plan	Number	IRR (95% CI)	*P*- value
No delay	670	reference	
60 days to < 2 years	600	1.2 (1.07–1.42)	0.004
2 to < 4 years	317	1.3 (1.15–1.58)	0.000
4 years and over	915	2.6 (2.28–3.08)	0.000
Sex (men v women)		1.1 (1.01–1.13)	0.024
Age group (ref: 15–24 years)		1.0 (1.00–1.00)	0.17
Period before e-records		2.0 (1.78–2.29)	0.000

## Discussion

Two previous studies involving remote Australian Aboriginal populations have demonstrated associations between increased service utilisation and health outcomes. A study in Western Australia demonstrated a positive association between the re-organisation of health services, increased utilisation and improved health outcomes, [27] while an NT study reported that an optimal level of primary care utilisation was associated with lower levels of hospital admissions, including for diabetes. [8] Our study steps beyond measuring utilisation by assessing a quality of service measure: the delay between a diagnosis of diabetes and the documentation of a diabetes care plan and finds that a delay beyond 60 days is associated with poorer diabetes control and an increase in hospital admissions. A study in the US provides a possible explanation for the pathway of this improvement where a comprehensive patient management program focusing on regular HbA1C tests, foot and eye care, and cholesterol screening improved the clinical outcome including reduced hospital admissions and hospital bed days. [17] Other studies have also found that better managed primary care was associated with increased disease monitoring activities, short term improvement in blood glucose control, reduced diabetes complications, reduction in diabetes-related hospital admissions, as well as significant reduction in health care costs. [9, 13, 16–19] The implication of the results in our study is that among those patients with care plans, a timely diabetes care plan is associated with more active clinical management compared with those for whom there is delay.

Our study did not observe an association between the early development of a care plan and attaining optimal blood pressure control in patients with diabetes [28]. This may be due to relatively small numbers of patients with hypertension in the study group, however the result is consistent with a previous study. [29] Optimal blood pressure control consists of several key elements, including regular physical activity, weight control, an appropriate drug regime for treatment and compliance with treatment and regular monitoring of the blood pressure. [28–30] More vigorous blood pressure control measures need to be employed to prevent the progression of vascular disease. A positive result from our study was the reduction in risk for diabetes-related hospital admissions for those patients diagnosed after the implementation of electronic health records. While not an aim of this study, this finding is consistent with the benefit of automated reminders and decision support available with electronic records.

Half of the study population had no documentation of a diabetes care plan two years after diagnosis suggesting there are barriers for recommended care for these patients. The NT has an extensive network of health centres in remote Aboriginal communities, all of which have standard protocols for the management of chronic disease and with the additional benefit of shared electronic health records. [9, 10, 24] The NT Aboriginal population is highly mobile and the shared health records mean that a patient’s medical history including current medications and results of recent pathology are available at any of the network of health centres. Recall systems are also available which are managed by local health care workers and supported by diabetes specialist outreach service and have been reported to achieve significant improvement in health outcomes. [31] However other barriers remain that limit optimal health care. These barriers include staff shortages and high staff turnover, which affects both the availability and training of the health staff and their acceptability to their Aboriginal clients. [32]

The strength of our study is the ability to use both routinely collected primary care and hospital data to examine a primary care intervention and the related health outcomes in a large population cohort. There are a number of limitations of the study. Firstly, electronic primary care records were implemented relatively recently and required historical paper records to be manually entered. The completeness of this historical data is anecdotally reported as reliable but has not been formally assessed and diabetes cases from before 2011 may have been under-reported. This shortcoming was offset by the inclusion of hospital admission data as a supplementary data source and also by the use of a flag, in analysis, for a diagnosis either before or after PCIS implementation. A second limitation is that the onset of diabetes is insidious and the true date of onset may not be known. This limitation is addressed in this population by the active screening programs including annual adult health checks that incorporate screening for diabetes. This study used the date of earliest recorded diagnosis in two data sources (PCIS and HSD) to improve the accuracy of the date of disease onset. A third limitation is whether the results can be generalised. This study involved a substantial but select Aboriginal population living in remote NT communities. There are similar populations living in other parts of Australia for whom the results may be directly applicable however caution should be taken against generalising to the broader population. A final limitation is that that there may be unmeasured factors that influence the outcomes. These may include risks such as variations in socioeconomic status of clients and quality and access of health services. While the benefits of patient-specific care plans are being reported in many different settings the more specific benefit of early implementation of care plans will need to be repeated in other populations to assess whether the results in this study are more generally applicable.

The progress of diabetes and associated complications can be attenuated by appropriate management [32, 33]. This study adds to the existing literature with encouraging evidence for the association between early introduction of planned care and improved glycaemic control. The study also identified that there were substantial delays in the implementation of diabetes care plans for many patients in the study population. This may reflect continuing gaps in delivering a suitable model of care to the service population. Timely diagnosis and an appropriate management plan, optimal clinical treatment, and motivated self-care are all key steps to improved diabetes management and improved quality of life for the NT Aboriginal population.

## Conclusion

The study found that a timely diabetes care plan was associated with better short-term blood glucose control and fewer diabetes-related admissions. The results highlighted the importance of early development of a comprehensive diabetes care plan. Delays may be a result of both patient and service-related factors.

## Additional file

Additional file 1: **Table S1.** Number of patients included in the study, NT remote Aboriginal community, 2008–2013. (DOCX 13 kb)

## Data Availability

The datasets generated and/or analysed during the study are not publicly available due to identifiability of remote primary care providers and the requirement to protect their privacy.

## Abbreviations

95% CI: 95% confidence interval
BP: Blood pressure
GP: General Practitioner
HbA1C: Refers to glycated haemoglobin. It develops when haemoglobin, a protein within red blood cells that carries oxygen throughout your body, joins with glucose in the blood, becoming ‘glycated’
HRN: Hospital Registration Number
HSD: Hospital Separations Dataset
ICD-10-AM: International Classification of Disease 10th Version, Australian Modified
ICPC-2-R: International Classification of Primary Care, revised second edition
IRR: Incidence Rate Ratio
NT: Northern Territory
PCIS: The Primary Care Information System
