# Supplementary material for: Does delay in planned diabetes care influence outcomes for aboriginal Australians? A study of quality in health care
Source: BMC Health Serv Res. 2019 Aug 19;19:582. doi: 10.1186/s12913-019-4404-7 (PMC6699070; doi:10.1186/s12913-019-4404-7)
Supplement: Supplementary file 1 — Table S1. Number of patients included in the study, NT remote Aboriginal community, 2008–2013. (DOCX 13 kb) [file 12913_2019_4404_MOESM1_ESM.docx]

**Additional file 1.**

**Table A1. Number of patients included in the study, NT remote Aboriginal community, 2008-2013**

| **Age group (years)** | **Men** | **Wo men** | **Total** |
| --- | --- | --- | --- |
| 15- 24 years | 32 | 133 | 165 |
| 25- 34 years | 208 | 392 | 600 |
| 35- 44 years | 325 | 480 | 805 |
| 45- 54 years | 237 | 349 | 586 |
| 55- 64 years | 101 | 199 | 300 |
| 65 and over | 43 | 68 | 111 |
| Total | 946 | 1,621 | 2,567 |
